# Supplementary material for: The History, Efficacy, and Safety of Potential Therapeutics: A Narrative Overview of the Complex Life of COVID-19
Source: Int J Environ Res Public Health. 2021 Jan 22;18(3):955. doi: 10.3390/ijerph18030955 (PMC7908443; doi:10.3390/ijerph18030955)
Supplement: Supplementary file 1 [file ijerph-18-00955-s001.pdf]

Supplementary Material

3.1. Group A: Inhibitors of SARS-CoV-2 Replication

3.1.1. Remdesivir

Table S1:

Reference	Type of Study Design	Clinical Evidence of Effectiveness in COVID-19 Patients	Clinical Evidence of Safety in COVID-19 patients	
			Adverse Events of Any Grade	Withdrawals
**Hillaker et al. [22]**	Case report	Effective	Not Disclosed	N/A*
**Antinori et al. [23]**	Prospective (compassionate), open-label study	Effective	Reported	Reported
**Grein et al. [24]**	Open-label cohort study	Effective	Reported	Reported
**Beigel et al. [25]**	Double-blind, randomized, placebo-controlled trial	Effective	Reported	Reported
**Wang et al. [26]**	Randomized, double-blind, placebo-controlled, multicenter trial	Not Effective	Reported	Reported

* N/A: Not applicable

3.1.2. Favipiravir

Table S2:

Reference	Type of Study Design	Clinical Evidence of Effectiveness in COVID-19 Patients	Clinical Evidence of Safety in COVID-19 patients	
			Adverse Events of Any Grade	Withdrawals
**Cai et al. [30]**	Open-Label control study	Effective	Reported	Not Reported
**Chen et al. [31]**	Prospective, randomized, controlled, open-label multicenter trial	Not Effective	Reported	Not Reported

* N/A: Not applicable

3.2. Group B: Inhibitors of SARS-CoV-2 Entry

3.2.1. Hydroxychloroquine

Table S3:

**Reference**	**Type of Study Design**	**Clinical Evidence of Effectiveness in COVID-19 Patients**	**Clinical Evidence of Safety in COVID-19 patients**	
			Adverse Events of Any Grade	Withdrawals
**Gautret et al. (a) [41]**	Open-label non-randomized clinical trial	Effective	Not Disclosed	Not Disclosed
**Gautret et al. (b) [42]**	Pilot observational study	Effective	Reported Abnormal ECG Findings	Not Disclosed
**Arshad et al. [43]**	Multi-center retrospective observational study	Effective	No Abnormal ECG Findings	Not Disclosed
**Rosenberg et al. [44]**	Retrospective cohort study	Not Effective	Reported Abnormal ECG Findings	Not Disclosed
**Mahévas et al. [45]**	Observational comparative study	Not Effective	Reported Abnormal ECG Findings	Reported
**Mehra et al. [46]**	Multinational registry analysis	Not Effective	Reported Abnormal ECG Findings	Not Disclosed
**Geleris et al. [47]**	Observational study	Not Effective	Reported Abnormal ECG Findings	Not Disclosed
**Tang et al. [48]**	Multicenter, open label, randomized controlled trial	Not Effective	No Abnormal ECG Findings	Reported
**Self et al. [49]**	Multicenter, blinded, placebo-controlled randomized trial	Not Effective	Reported Abnormal ECG Findings	Not Disclosed

* N/A: Not applicable

3.3. Group C: Adjunctive Therapies

3.3.1. Tocilizumab

Table S4:

**Reference**	**Type of Study Design**	**Clinical Evidence of Effectiveness in COVID-19 Patients**	**Clinical Evidence of Safety in COVID-19 patients**	
			Adverse Events of Any Grade	Withdrawals
**Alattar et al. [63]**	Retrospective study	Effective	Reported	Not Reported
**Capra et al [64]**	Retrospective observational study	Effective	Not Disclosed	Not Disclosed
**Di Giambenedetto et al. [65]**	Case series	Effective	Not Reported	Not Disclosed
**Klopfenstein et al. [66]**	Retrospective case-control study	Effective	Not Disclosed	Not Disclosed
**Luo et al. [67]**	Single center retrospective study	Effective	Not Disclosed	Not Disclosed
**Mazzitelli et al. [68]**	Case series	Effective	Reported	Not Disclosed
**Quartuccio et al. [69]**	Single center retrospective study	Effective	Not Reported	Not Disclosed
**Sciascia et al. [70]**	Pilot prospective open, single-arm multicenter study	Effective	Not reported	Not Disclosed
**Toniati et al. [71]**	Single center prospective study	Effective	Reported	Not Disclosed
**Xu et al. [72]**	Retrospective study	Effective	Not Reported	Not Disclosed
**Cellina et al. [73]**	Case report	Effective	Not Disclosed	N/A
**De Luna et al. [74]**	Case report	Effective	Not Disclosed	N/A
**Michot et al. [75]**	Case report	Effective	Reported	N/A
**Wang et al. [76]**	Case report	Effective	Not Reported	N/A
**Campochiaro et al. [77]**	Single-center retrospective cohort study	Not Effective	Reported	Not Disclosed
**Colaneri et al. [78]**	Controlled retrospective study	Not Effective	Not Reported	Not Disclosed
**Morena et al. [79]**	Open-label prospective study	Not Effective	Reported	Not Disclosed
**Radbel et al. [80]**	Case report	Not Effective	Reported	N/A

3.3.3. Convalescent Plasma

Table S5:

Reference	Type of Study Design	Clinical Evidence of Effectiveness in COVID-19 Patients	Clinical Evidence of Safety in COVID-19 patients	
			Adverse Events of Any Grade	Withdrawals
**Ahn et al. [88]**	Case series	Effective	Not Reported	Not Disclosed
**Çınar et al. [89]**	Case report	Effective	Not Reported	N/A
**Duan et al. [90]**	Case series	Effective	Reported	Not Disclosed
**Kong et al. [91]**	Case report	Effective	Not Disclosed	N/A
**Salazar et al. [92]**	Case series	Effective	Not Reported	Not Disclosed
**Ye et al. [93]**	Case series	Effective	Not Reported	Not Disclosed
**Zeng et al. [94]**	Case series	Not Effective	Not Reported	Not Disclosed
**Li et al. [95]**	Open-label, multicenter, randomized clinical trial	Not Effective	Reported	Not Reported
